# Isolated Fracture of the Acromion Process: A Case Report

**DOI:** 10.7759/cureus.14032

**Published:** 2021-03-22

**Authors:** Indranil Bhaduri, Rajesh Thakur, Sachin Kumar, Manoj K Rajak

**Affiliations:** 1 Department of Joint Replacement and Orthopedics, Tata Main Hospital, Jamshedpur, IND

**Keywords:** surgical case report, acromion process fracture, late surgical intervention, constant score

## Abstract

Fracture of the acromion process is an uncommon injury which is often diagnosed late. Though, usually managed conservatively, the indications for surgery in these fractures are very specific. A 52-year-old active man attended the out-patient department of our hospital following an injury to the right shoulder. An X-ray revealed a Type II, minimally displaced fracture of the base of the acromion process. Conservative management was attempted initially, which was converted to surgical stabilization after six weeks when it was noticed that the fracture had failed to unite and had progressed to become a displaced Type III fracture. Post-operative period was uneventful with a gradual return to the pre-injury level of function of the right shoulder, which was assessed by the Constant Score as well as the University of California Los Angeles (UCLA) shoulder score. The satisfaction with the final functional outcome was assessed by the UCLA shoulder score. Clinicians must look actively for acromion process fractures in all shoulder injuries. Minimally displaced fractures should be regularly followed up for displacement and sub-acromial space compromise. Although acromion fractures are usually treated conservatively, albeit a higher non-union rate, they should be treated surgically in the event of displacement or sub-acromial space reduction, in order to achieve good functional recovery.

## Introduction

Fractures of the acromion process of the scapula are extremely rare, comprising only 3%-5% of all shoulder injuries and about 7%-8% of scapular fractures [1]. This fracture has seen a renewed interest among orthopedic trauma surgeons in recent times [2]. This has been mainly because of the functional compromise these injuries cause due to the shoulder impingement concomitant to a reduced sub-acromion space seen in untreated displaced fractures [3-6]. Also, the propensity of undisplaced acromion process fractures to displace, over time, due to the weight of the suspended upper limb might result in sub-acromion space compromise [7]. This case report was an attempt to present the functional outcome of operative treatment of an isolated minimally displaced acromion process fracture following 16 months of follow up after failure of attempted conservative management for six weeks.

## Case presentation

**Figure 1:**
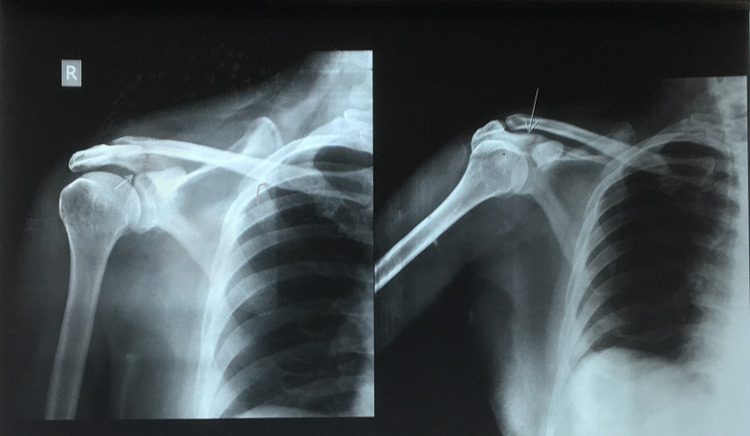
X-ray at presentation

A 52-year-old male, right hand dominant, sustained an injury to the right shoulder from a fall due to slipping at home and presented with pain and mild swelling and ecchymosis over the right shoulder, and associated minor abrasions over the forehead and right elbow. Both active and passive shoulder movements was painful. There was associated local tenderness and crepitus over the region of the acromion process of the right shoulder. There was no evidence of neurovascular compromise. Radiograph of the right shoulder revealed a minimally displaced fracture of the acromion process, Type II according to Kuhn classification, at the junction of the spine of the scapula and the process (Figure 1).

**Figure 2:**
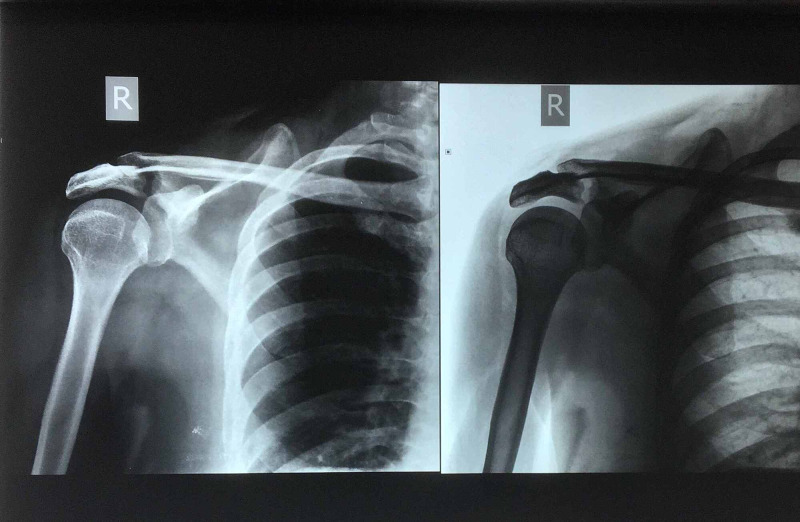
X-ray at five weeks showing displacement and subacromial space compromise

It was decided to treat the fracture along conservative lines with regular follow up for at least six weeks. with conversion to surgical stabilization if so required. Conservative treatment was done with a commercially available shoulder immobilizer and arm-pouch. Since these fractures are known for their propensity to displace, serial X-rays were taken after a week and thereafter at weekly intervals up to five weeks, for evidence of healing as well as displacement, if any. At the end of five weeks, an increase in the fracture gap and inferior displacement of the acromion, compromising the sub-acromial space was noticed; a progression to Type III, and the decision to operatively stabilize the fracture was made (Figure 2).

The patient was prepared for surgery after routine blood investigations and mandatory screening for human immunodeficiency virus (HIV), hepatitis C virus (HCV), and hepatitis B surface antigen (HBsAg). He underwent a pre-anesthesia check-up for assessment of fitness for surgery.

Under general anesthesia and prone position, the fracture was approached with an incision placed directly over the spine of the scapula, curving anteriorly. No deltoid or rotator cuff tear observed during the surgery. The fracture was identified and was found to be separated by a gap of more than 5 millimetres. The acromion process fragment was augmented, and the fracture was reduced under direct vision. After temporary stabilisation with two smooth Kirschner wires under image guidance, the fracture was stabilized with a 6-hole, 3.5-mm locking reconstruction plate, in compression mode, using two cortical screws and four locking bolts.

**Figure 3:**
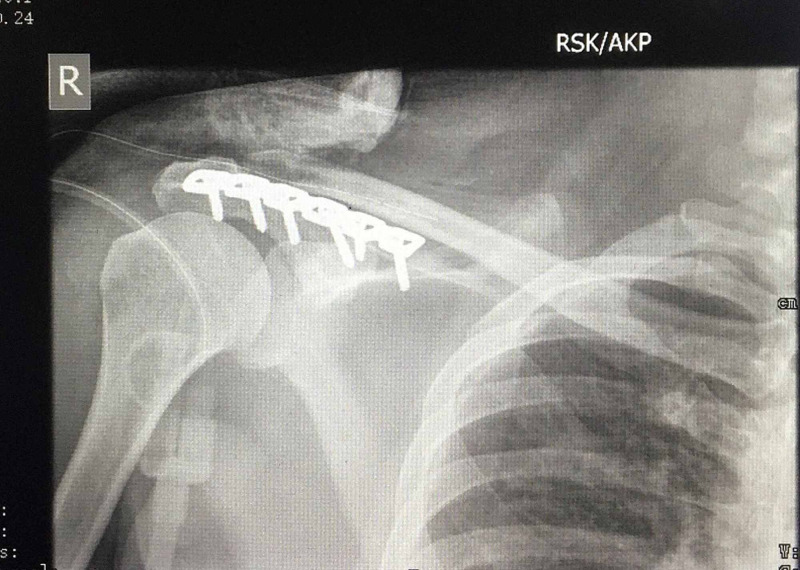
Immediate post-operative film

**Figure 4:**
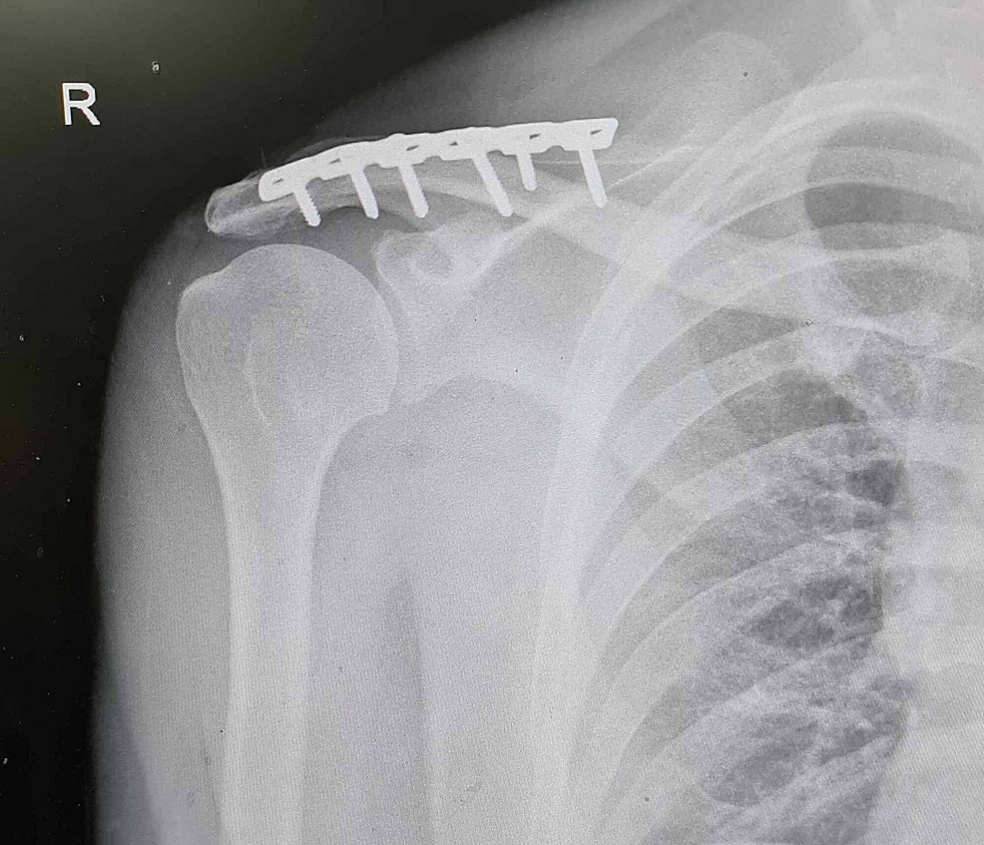
At 12 weeks; union complete

Post-operative period was uneventful. Operative wound was examined and dressed after forty-eight hours, with drain removal at the same time, according to our departmental protocol. Gentle active movements and progressive exercises was initiated one week after the surgery within the limits of pain tolerance. Radiographs were taken 24 hours after the surgery (Figure 3). They were then taken at six weeks, 12 weeks which were found to be acceptable with a clear sub-acromial space. Bony union was evident by 12 weeks (Figure 4).

**Figure 5:**
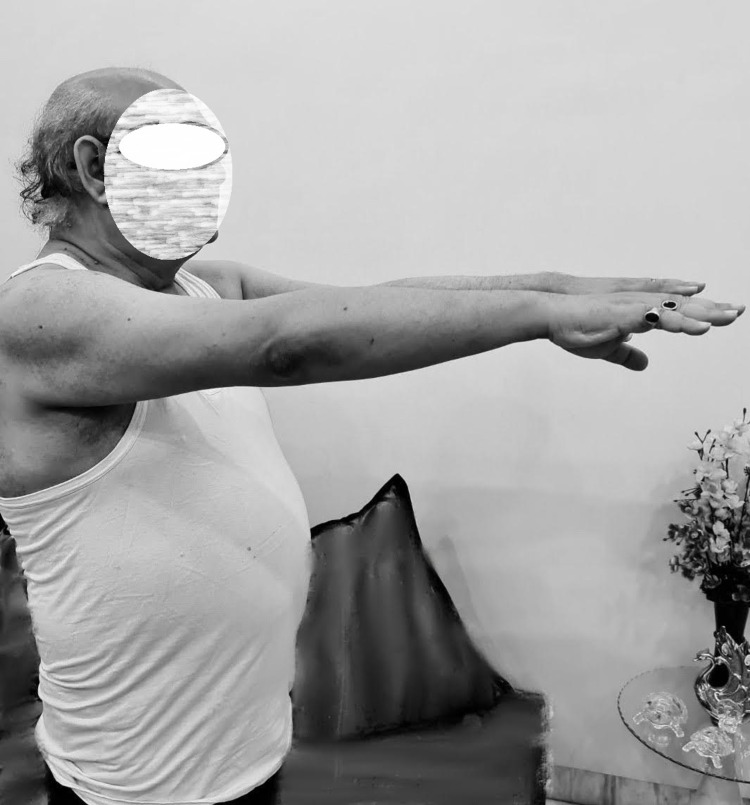
Full range of movements at final follow up

**Figure 6:**
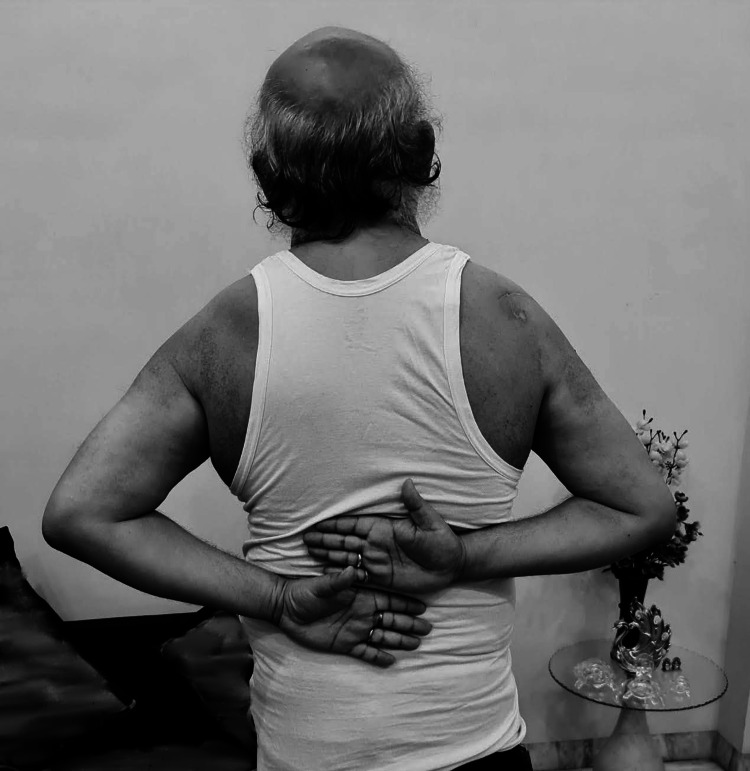
Full range of movements at final follow up

**Figure 7:**
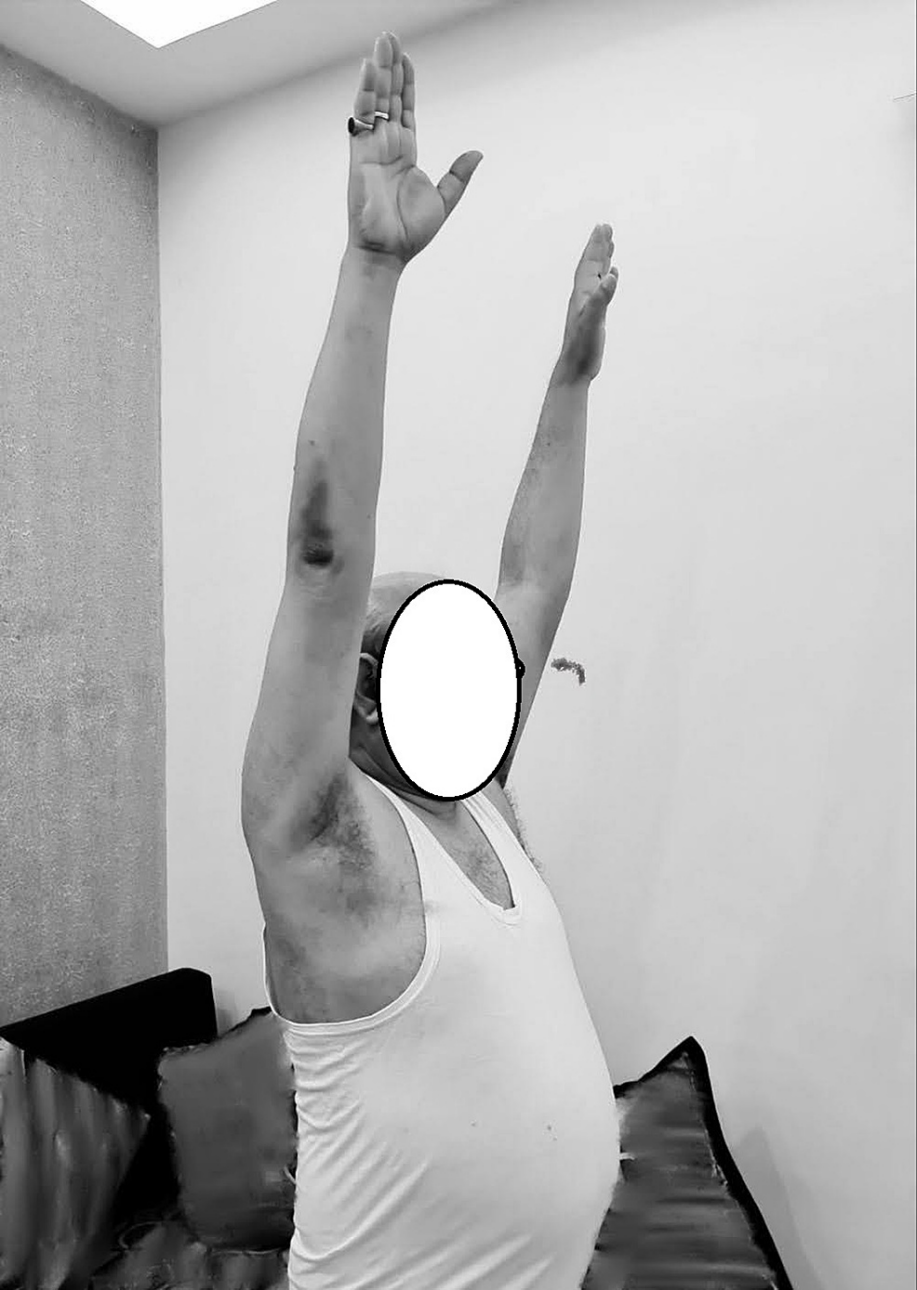
Full range of movements at final follow up

The functional outcome was assessed using the University of California Los Angeles (UCLA) Shoulder Function Scale and the Constant (Murley) Score at six weeks, 12 weeks, six months and 16 months. The score was 9 at six weeks, 15 at three months, 28 at six months and 33 at 16 months with the UCLA Shoulder Score and 33% at six weeks, 62% at three months, 82% at six months and 98% at 16 months according to the Constant Score. At the final examination, at 16 months, the patient was fully satisfied with good functional range of movement (Figures 5-7).

## Discussion

The scapula is anatomically positioned at the posterosuperior border of the thorax, attached by several muscles to the pectoral girdle, and plays an important part in the biomechanics of the upper limb [8]. 

We used the Kuhn classification for acromion fractures and our case was classified as Kuhn Type II which subsequently progressed to Type III, when operative intervention was decided [9]. Isolated fracture of the acromion process following direct trauma is a very uncommon entity, though they have been reported with other associated injuries and fractures around the shoulder girdle [10]. Although many of these fractures heal uneventfully, the worst outcomes have been reported with fractures that occur where the acromion base attaches to the scapular spine [11]. Our case had a fracture at this site. For fractures around the shoulder, a radiological trauma series is performed with AP, axillary, and profile projections of the shoulder. Often, a CT Scan with 3D reconstruction is very helpful in the diagnosis and planning of treatment of acromion process fractures and should form an integral part of the diagnostic work-up [12]. In our case, only an X-ray assessment was done. The primary objectives of open reduction and internal fixation (ORIF) are anatomical repositioning of the lateral fragment to restore the physiological width of the sub-acromial space, to establish a rigid fixation to neutralize the deltoid muscle forces, and to provide sufficient compression on the fracture for proper bone healing.

Kuhn has advocated surgical stabilization of Type III fractures that compromise the sub-acromial space, symptomatic stress fractures, and painful non-unions [9]. Depending on age, activity, and general condition of the patient, internal fixation is recommended in grossly displaced fractures of the acromion and coracoid process as concluded by Bauer et al. [13]. Hess et al. also concluded in their study that patient characteristics, such as activity level, might be a relevant parameter when selecting a treatment strategy. Early fixation may be the most sensible way to treat working adults who need to avoid long absences from work [14]. Our patient was an active middle-aged man with field job, so we decided to operate after giving conservative treatment a fair trial. Hill and his co-workers advocated surgical intervention for sub-acromial impingement, symptomatic non-unions, open fractures, displacement of more than 1 cm, and disruption of the superior shoulder suspensory complex [15]. Acromion fractures, based on fracture configuration, have been surgically treated with implants ranging from cancellous screws [7,15], narrow 3.5 mm dynamic compression plates and cortical screws [15], and locking plates and screws [16] plain or threaded Kirschner wires [17,18] and tension band [19]. Fixation with K-wires is not recommended because it may cause early implant failure and stable reconstruction may not be achieved after surgery [15]. Although acromion fractures have been fixed with pre-bent clavicle reconstruction plates [18], we decided to stabilize the fracture with a 3.5 mm locked reconstruction plate in compression mode to obtain a stable construct. We did not observe any complications related to either the operative procedure or the implants used, in our case. Postoperative recovery was uneventful. Functional recovery was assessed using the UCLA Shoulder Function Scale and the Constant (Murley) Score and compared to tabulations at the end of each follow up to record improvement.

## Conclusions

A high degree of awareness must be exhibited by the clinician while evaluating a patient with shoulder trauma, who should be carefully examined for possible scapular process fractures. Although fractures of the acromion process are commonly treated conservatively, surgery should be offered to patients who show features of impingement and radiological evidence of compromise of the sub-acromial clear space. The non-union rate with conservative treatment, although relatively high, is often not overtly painful nor is it limiting to reasonable shoulder function, especially in the elderly or less active patients. Surgical stabilization appears to be a more suitable option of treatment for physically active patients who are more likely to present with symptomatic non-unions. In this paper, we report an uncommon case of an isolated traumatic acromion process fracture which became displaced during the course of conservative management and the treatment strategy had to be revised to surgical stabilization. Despite the delay in surgical stabilization, the functional outcome was good.
